# Inflammatory neuropathy with evidence of anti-GQ1b antibodies in angioimmunoblastic T cell lymphoma (AITL): a case report

**DOI:** 10.1007/s00415-024-12217-3

**Published:** 2024-02-08

**Authors:** Antonia Kleeberg, Gregor Bauer, Erik Jung, Jan C. Purrucker

**Affiliations:** Department of Neurology, Heidelberg University Hospital, Im Neuenheimer Feld 400, 69120 Heidelberg, Germany

## Medical history and clinical findings

A 66-year-old male patient suspected of having Guillain-Barré syndrome (GBS) was referred to us by the internal medicine department of a peripheral hospital. The patient had developed rapidly progressive tetraparesis, in particular with leg pareses, and urinary and fecal incontinence. Subacute symptoms had started 2 weeks earlier accompanied by excruciating whole-body pain that the patient had noticed when he woke up.

On admission to our neurological intermediate care unit, the patient presented with paraplegia and symmetrical, distally concentrated paresis of the arms with strength grade 3–4/5 on the Medical Research Council (MRC) muscle scale. In addition, we found areflexia of the lower extremity and reflex attenuation of the upper extremity. The patient did not report new sensory disturbances. No abnormalities were discovered in the cranial nerve examination. Physical examination revealed several palpable but indolent inguinal, submandibular and preauricular lymph nodes. Furthermore, a confluent macular exanthema was present, and the patient was dyspneic with known bilateral pleural effusions.

Regarding the patient's medical history, he had been diagnosed with Ann-Arbor stage IIIS B angioimmunoblastic T cell lymphoma (AITL) 5 months previously (see text box). The patient had received four cycles of CHOP chemotherapy (cyclophosphamide 750 mg/m^2^, hydroxydaunorubicin 50 mg/m^2^, vincristine 1,4 mg/m^2^ and prednisone) to date, in the last cycle at reduced doses and without vincristine due to polyneuropathy common toxicity criteria (CTC) II°. The last chemotherapy cycle had taken place about 1 month prior to admission to our department. The last tumor staging with a CT scan including neck and torso had been performed one and a half months prior to admission and had shown an improvement in findings compared to the initial tumor staging. In addition, the patient was known to have had bladder carcinoma that was first diagnosed 6 years earlier and treated with intravesical instillation of mitomycin C. Other than that, the patient’s medication intake included apixaban (due to atrial fibrillation), bisoprolol, ramipril, torasemide, pantoprazole and folic acid.

Angioimmunoblastic T-cell lymphoma (according to [1])

- Age peak 60–70 years, m > w (slight predominance)
- Rapidly progressive
- Characterized by general lymphadenopathy often associated with fever and weight loss as well as with hepato- and/or splenomegaly
- Accompanied by multiple immune disorders (hypergammaglobulinemia, hemolytic anemia, autoantibodies, cryoglobulins, or cold agglutinins, …)
- Up to 70% of cases with bone marrow involvement
- Up to 50% with skin rash
- Pathomechanism: follicular T-helper cells stimulate B cells which turn into plasma cells that produce excessive amounts of antibodies

## Diagnostic procedures

The first cerebrospinal fluid (CSF) analysis obtained 14 days after symptom onset showed lymphocytic pleocytosis with 11 cells/µl and normal protein. As systemic herpes simplex virus (HSV) infection (positive HSV-IgM in serum) had been suspected, aciclovir therapy had already been started in the primary hospital; we initially continued that treatment under the differential diagnosis of a HSV meningoradiculitis, which was eventually ruled out by polymerase chain reaction (PCR) and serological testing.

Blood analysis showed a vitamin B6 deficiency which we supplemented orally.

**Fig. 1:**
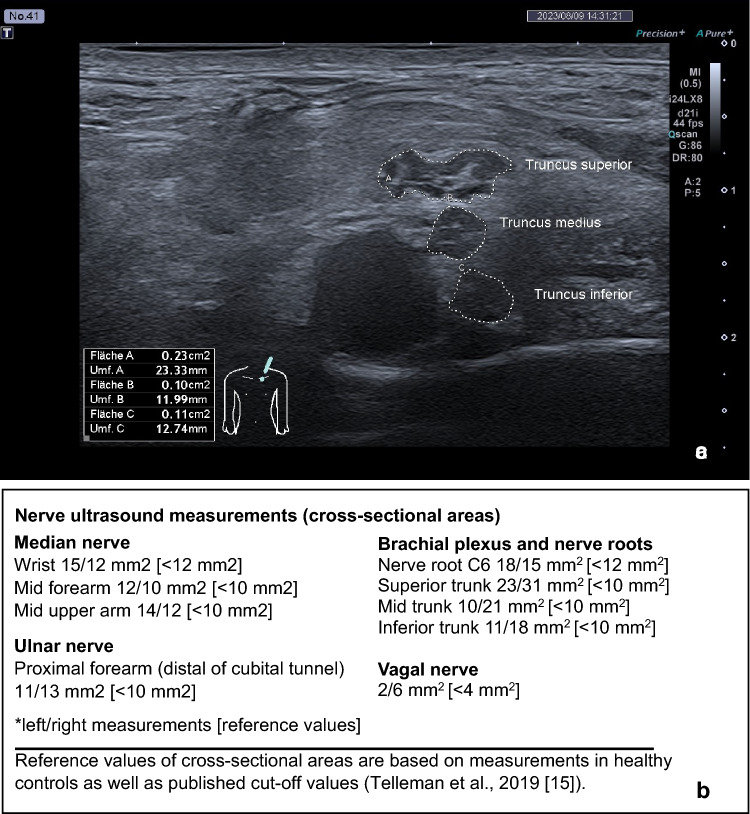
Nerve ultrasound examination. **a** Exemplary ultrasound image (Canon Applio i700; 21 MHz probe) of the left brachial plexus demonstrating enlargement of brachial plexus at the level of the trunks (Fläche = area; Umf. = Umfang = circumference). **b** Nerve ultrasound measurements [reference values in brackets]

Contrast-enhanced spinal MRI (15 days after symptom onset) showed unremarkable findings. Electroneurography (15 days after symptom onset) revealed severe sensorimotor polyneuropathy that could not be reliably classified into an axonal or demyelinating form due to its severity. Electromyographically, there were signs of both chronic and florid denervation. Nerve sonography showed diffuse symmetrical enlargement of nerve cross-sectional areas in the arms, the brachial plexus and nerve roots bilaterally, consistent with an inflammatory polyneuropathy (such as acute or chronic inflammatory demyelinating polyradiculoneuropathy; AIDP or CIDP; Fig. 1).

In the absence of detecting any pathogen in the CSF obtained initially (HSV, varicella zoster virus, Tick-borne encephalitis virus, enterovirus, borrelia, universal fungal PCR and eubacterial PCR all negative) and limitations to cytologically assessing that sample, a new lumbar puncture including immunocytological analysis was performed a few days later. Aciclovir therapy was discontinued.

The follow-up lumbar puncture (16 days after symptom onset) showed lymphocytic pleocytosis again (16 cells/µl), still without protein levels being elevated. Finally, immunocytological analysis (flow-cytometric analysis, non-Hodgkin lymphoma minimal residual disease panel; TCR (CD3/CD4/CD8) and BCR (CD19/CD20) as well as CD45/kappa/lambda were examined) showed both clonal B cell and T cell populations in serum and CSF. Cytologically, lymphomatous meningitis was now suspected; therefore, we added a cranial MRI (23 days after symptom onset), which showed no signs of solid meningeosis or other lymphomatous manifestation.

Oligoclonal bands were positive, showing both pattern 4 (identical bands in serum and CSF, arguing for a systemic inflammatory response) and pattern 5 (showing monoclonal bands in CSF and serum, indicative of systemic gammopathy) [18].

**Fig. 2:**
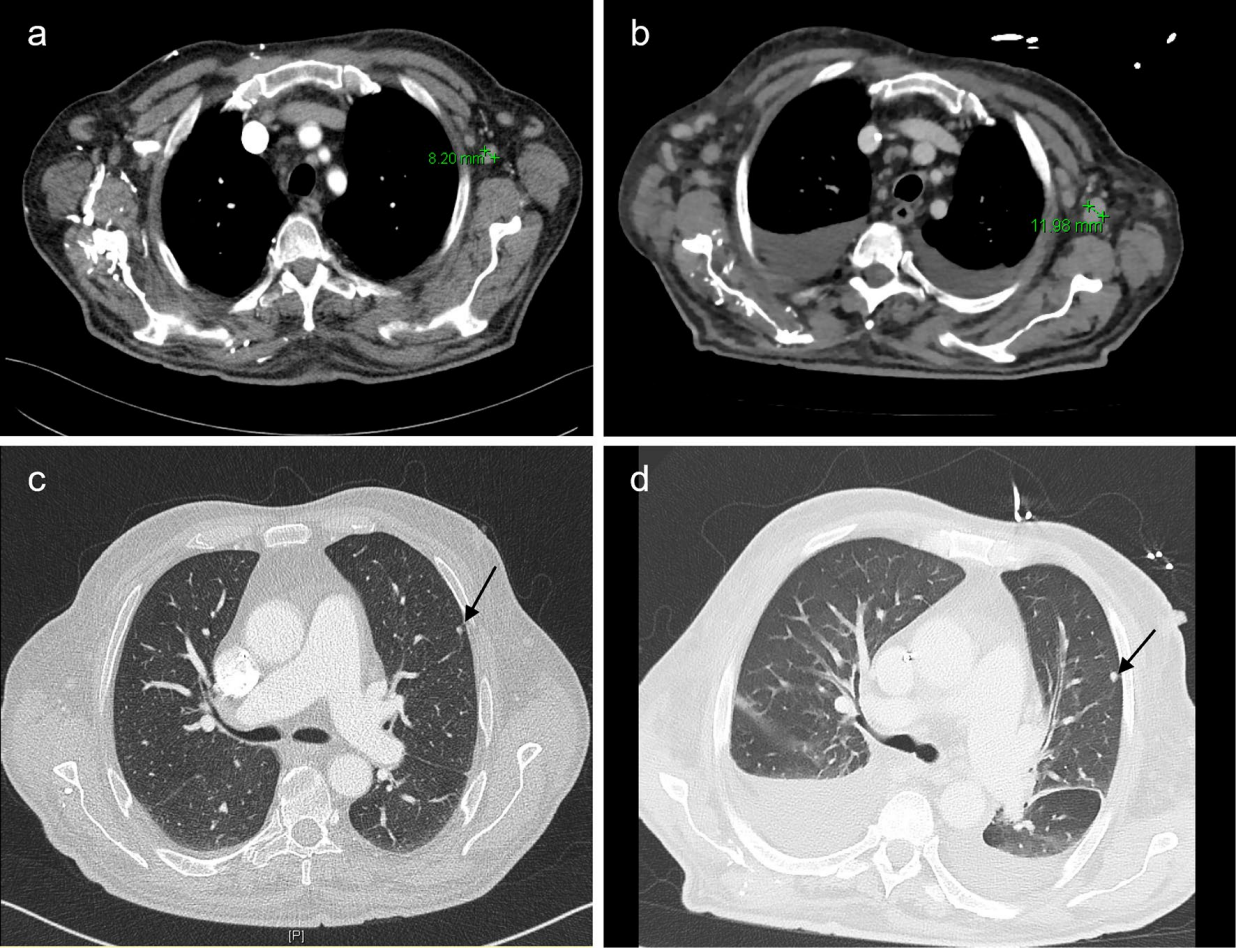
Selected sections from the patient's staging CTs in 04/2023 and 06/2023. **a** Axillary lymph node 04/2023, approx. 8 mm in diameter. **b** Axillary lymph node 06/2023, approx. 12 mm in diameter. **c** Pulmonary round nodule 04/2023, approx. 4 mm in diameter. **d** Pulmonary round nodule 06/2023, approx. 6 mm in diameter

We also performed a CT examination of the neck/thorax/abdomen/pelvis (17 days after symptom onset) to determine whether the known angioimmunoblastic T cell lymphoma had progressed. The scans showed several lymph nodes and pulmonary round nodules to be progressing in size or newly developed compared with the previous tumor-staging CT (Fig. 2). We thus suspected progression of the patient’s known lymphoma. The volume of the pleural effusions was also increasing compared to the last CT. Soluble interleukin-2 receptor was significantly elevated at > 5000 U/ml as an additional activity marker of lymphoma.

As paraproteinemia had been suspected from the CSF diagnostic tests, we supplemented the procedures with serum and urine electrophoretic studies as well. They revealed an M-spike, also detecting monoclonal IgG type kappa and lambda as well as monoclonal IgM type kappa in serum and Bence-Jones protein type kappa in urine.

Interestingly, in the extended serological antibody diagnostics, GQ1b IgM antibodies were strongly positive (+ +  + / +  + +) in a membrane-based immunoblot assay. Ri- and SOX1 IgG antibodies were only marginally elevated (( +)/ +  + +). Other anti-neuronal antibodies were negative, including anti-neurofascin 155 and 186.

## Therapy and further course

Under probationary cortisone pulse therapy with 1 g methylprednisolone for 5 days (starting on day 18 after symptom onset) owing to the suspected paraneoplastic inflammatory polyneuropathy, the strength in the legs improved slightly to strength grade 1–3/5 on the MRC scale, but then deteriorated again. Furthermore, the exanthema improved following the prednisolone therapy. We then administered intravenous immunoglobulin for 5 days (starting on day 23 after symptom onset). Following this therapy, the strength in the legs again improved somewhat to strength grade 1–2/5 on the MRC scale.

While the patient was still receiving intravenous immunoglobulin therapy, we transferred him to our Clinic for Hematology and Oncology for further tumor-specific treatment. The known lymphoma suspected to be progressing, this time with bone marrow infiltration, was confirmed. Immunohistochemically, however, the cells were negative for surface markers; therefore, targeted therapy could not be carried out. Due to the patient’s markedly reduced general condition, intensive chemotherapy and subsequent stem cell transplantation were not possible either. Therefore, palliative therapy with bendamustine to control the symptoms in the presence of highly suspected pulmonary infiltrates of the lymphoma was initiated (32 days after symptom onset). Plasmapheresis, which we had recommended to further improve the paresis, could not be performed due to the patient’s poor general condition. Considering the overall situation, the patient was moved to a palliative care unit owing to renewed respiratory deterioration 36 days after symptom onset. Some weeks later, he was transferred to a hospice and again a few weeks later he passed away.

## Discussion

In summary, this patient suffered from polyneuropathy with an inflammatory pattern, as supported by nerve sonography, and with evidence of GQ1b antibodies and paraproteinemia. Based on the patient's history and the frequent occurrence of autoimmune phenomena in AITL, it is probable that the paraneoplastic syndrome developed as a result of the known lymphoma. Under immunomodulatory therapy, the patient's paresis improved slightly. Focal or segmental enlargements of nerve cross-sectional areas and a “fried-egg sign” (echogenic core and a hypoechoic vascularized peripheral zone) which have been described in neurolymphomatosis were absent [2, 3].

Diagnosing this patient was difficult for various reasons: First, pre-existing polyneuropathy due to neurotoxic chemotherapy is to be assumed according to the previous findings and the literature (especially vincristine [4]). To complicate matters, the patient also suffered from vitamin B6 deficiency, which might also cause polyneuropathy. Nevertheless, the clinical presentation with a subacute onset and rapidly progressive course weeks after the last chemotherapy with severe motor symptoms led us away from a pure toxic or metabolic neuropathy. Both—being predominant axonal neuropathies—could also not explain the enlargement of nerve cross-sectional areas [16, 17]. Furthermore, the disease presented clinically like GBS, but albuminocytologic dissociation was not observed even 3 weeks after symptom onset. Otherwise, elevated protein has only been found in 80% of GBS patients in the second week after onset of symptoms [5]. A POEMS syndrome (polyneuropathy, organomegaly, endocrinopathy, monoclonal protein and skin changes) was ruled out by the unremarkable levels of vascular endothelial growth factor (VEGF).

**Fig. 3:**
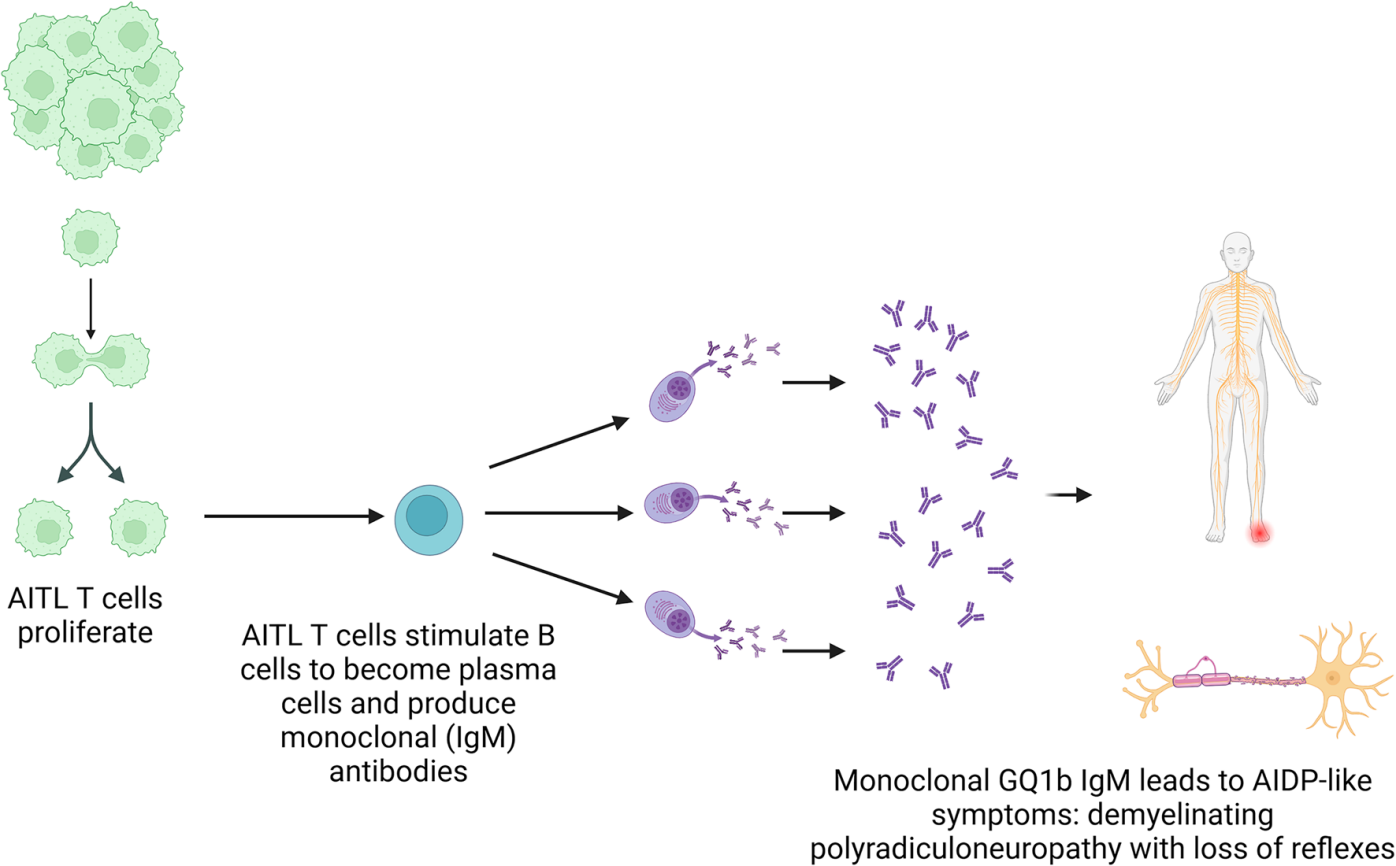
Suspected pathomechanism: Tumor T cells proliferate, stimulating a certain kind of B cell leading to proliferation of a plasma cell clone producing monoclonal antibodies, in our case GQ1b IgM antibodies, which in turn leads to AIDP-like symptoms (created with BioRender.com)

AITL is a very rare condition. According to the literature, monoclonal gammopathy occurs in 10–30% of all AITL cases [6, 7]. The pathomechanism suspected of linking these 2 conditions [1] is displayed in Fig. 3: The tumor follicular T-helper cells activate B cells, which turn into plasma cells and produce large amounts of antibodies. Furthermore, in several case reports AIDP has been linked to AITL [8, 9].

The peculiarity in our case is that certain autoantibodies, namely GQ1b IgM antibodies, were detected. These antibodies have been associated with neoplasia in individual case reports [10–12]. GQ1b antibodies are usually associated with Miller-Fisher syndrome, a variant of GBS that primarily affects the cranial nerves. In addition, this antibody is found in patients with Bickerstaff encephalitis. In particular, disease courses involving ophthalmoparesis are associated with GQ1b antibodies—but cranial nerve palsies were not present in our patient at any time. However, one case report has been published concerning a patient with CIDP-like symptoms and positive GQ1b antibodies in T cell lymphoma [13] and another on two patients with positive GQ1b antibodies, CIDP-like symptoms and IgM paraproteins [14].

In summary, the following link could be established: Following proliferation of T-helper cells, B cells were activated. A certain clone of plasma cells originated, producing GQ1b IgM. By mechanisms not yet known, this GQ1b paraproteinemia generated AIDP-like symptoms (Fig. 3).

With regard to a more efficient clinical work-up in patients with newly developed polyneuropathy and a history of lymphoma, clinicians might consider early performance of immunocytological and pathological assessment of the CSF as well as assessment of anti-neuronal/anti-ganglioside antibodies. Afterward, the more unspecific diagnostics including, e.g., nerve sonography could be carried out, before all diagnostic elements could be reviewed together.

Our case report has limitations. To gain more certainty regarding the etiology of this patient’s polyneuropathy, more examinations would have been helpful, such as a nerve biopsy or a nerve/plexus MRI. Unfortunately, due to the patient’s poor general condition which also deteriorated in the course of his hospitalization, these examinations were neither possible nor reasonable.

## Conclusion

Differentiating the cause of polyneuropathy in the patient reported here mainly constitutes an academic endeavor, considering the, unfortunately, unfavorable prognosis. Nevertheless, it shows the complex interplay of different factors in the development of polyneuropathy in lymphoma patients and the necessity to promptly initiate tumor-specific therapy if possible. It also highlights the importance of considering AITL in autoimmune phenomena as they can occur even before AITL is diagnosed.

## Data Availability

All data analyzed during this study are included in this published article.

## Abbreviations

AIDP: Acute inflammatory demyelinating polyradiculoneuropathy
AITL: Angioimmunoblastic T cell lymphoma
CIDP: Chronic inflammatory demyelinating polyradiculoneuropathy
CSF: Cerebrospinal fluid
GBS: Guillain-Barré syndrome
HSV: Herpes simplex virus
MRC scale: Medical Research Council scale
PCR: Polymerase chain reaction

## References

[1] De Leval L, Gisselbrecht C, Gaulard P (2010). Advances in the understanding and management of angioimmunoblastic T-cell lymphoma. Br J Haematol.

[2] Lee Y, Huang G, Chang W, Hsu Y (2021). Fried egg sign: a typical ultrasonography feature of neurolymphomatosis. J Clin Ultrasound.

[3] Wada A, Uchida Y, Hokkoku K, Kondo A, Fujii Y, Chiba T, Matsuo T, Tsukamoto H, Hatanaka Y, Kobayashi S, Sonoo M (2023). Utility of nerve ultrasound in the management of primary neurolymphomatosis: case report and review of the literature. Clin Neurophysiol Pract.

[4] Li G, Hu Y, Li D, Zhang Y, Guo H, Li Y, Chen F, Xu J (2020). Vincristine-induced peripheral neuropathy: a mini-review. Neurotoxicology.

[5] Van Der Meché FGA, Van Doorn PA, Meulstee J, Jennekens FGI (2001). Diagnostic and Classification Criteria for the Guillain-Barré Syndrome. Eur Neurol.

[6] Federico M, Rudiger T, Bellei M, Nathwani BN, Luminari S, Coiffier B, Harris NL, Jaffe ES, Pileri SA, Savage KJ, Weisenburger DD, Armitage JO, Mounier N, Vose JM (2013). Clinicopathologic characteristics of angioimmunoblastic T-cell lymphoma: analysis of the international peripheral T-cell lymphoma project. J Clin Oncol.

[7] Lachenal F, Berger F, Ghesquières H, Biron P, Hot A, Callet-Bauchu E, Chassagne C, Coiffier B, Durieu I, Rousset H, Salles G (2007). Angioimmunoblastic T-cell lymphoma: clinical and laboratory features at diagnosis in 77 patients. Medicine.

[8] Howell NA, Arya S, Tai PC, Sadeghian H, Sakhdari A, Wu R, Prica A (2022). Guillain-Barré syndrome as an early manifestation of angioimmunoblastic T-cell lymphoma. BMJ Case Reports.

[9] Pathak P, Perimbeti S, Ames A, Moskowitz AJ (2019). Guillain Barré syndrome heralding the diagnosis of angioimmunoblastic T-cell lymphoma. Leuk Lymphoma.

[10] Kloos L (2003). Paraneoplastic ophthalmoplegia and subacute motor axonal neuropathy associated with anti-GQ1b antibodies in a patient with malignant melanoma. J Neurol Neurosurg Psychiatry.

[11] Nokura K, Kako T, Azuma F, Samukawa M, Kusunoki S, Tanaka K (2020). Paraneoplastic cerebellar degeneration and limbic/brainstem encephalopathy associated with small cell lung cancer with serum positivity for anti-Hu and multiple anti-ganglioside (GM2, GQ1b, GaLNAc-GD1a, GT1a) antibodies: a case report. Neurol Neurosurg.

[12] Robinson R, Coebergh J, Abdel-Aziz K (2016). Paraneoplastic anti-GQ1b syndrome associated with diffuse large B-cell lymphoma. J Neurol Neurosurg Psychiatr.

[13] Salem B (2019). Chronic demyelinating polyneuropathy associated with anti-ganglioside GQ1b antibodies in peripheral T-cell lymphoma. Arch Hematol Case Rep Rev.

[14] Carpo M, Pedotti R, Lolli F, Pitrola A, Allaria S, Scarlato G, Nobile-Orazio E (1998). Clinical correlate and fine specificity of anti-GQ1b antibodies in peripheral neuropathy. J Neurol Sci.

[15] Telleman JA, Herraets IJT, Goedee HS, Verhamme C, Nikolakopoulos S, Van Asseldonk J-TH, Van Der Pol WL, Van Den Berg LH, Visser LH (2019). Nerve ultrasound: a reproducible diagnostic tool in peripheral neuropathy. Neurology.

[16] Grimm A, Heiling B, Schumacher U, Witte OW, Axer H (2014). Ultrasound differentiation of axonal and demyelinating neuropathies. Muscle Nerve.

[17] Zaidman CM, Al-Lozi M, Pestronk A (2009). Peripheral nerve size in normals and patients with polyneuropathy: an ultrasound study. Muscle Nerve.

[18] Pinar A, Tuncer Kurne A, Lay I, Acar NP, Karahan S, Karabudak R, Akbiyik F (2018). Cerebrospinal fluid oligoclonal banding patterns and intrathecal immunoglobulin synthesis: data comparison from a wide patient group. Neurol Sci Neurophysiol.

